# An Optimized High-Throughput Neutralization Assay for Hepatitis E Virus (HEV) Involving Detection of Secreted Porf2

**DOI:** 10.3390/v11010064

**Published:** 2019-01-15

**Authors:** Chang Liu, Wei Cai, Xin Yin, Zimin Tang, Guiping Wen, Charuta Ambardekar, Xinlei Li, Dong Ying, Zongdi Feng, Zizheng Zheng, Ningshao Xia

**Affiliations:** 1 State Key Laboratory of Molecular Vaccinology and Molecular Diagnostics, National Institute of Diagnostics and Vaccine Development in Infectious Diseases, School of Life Sciences, Xiamen University, Xiamen 361102, Fujian, China; changl@stu.xmu.edu.cn (C.L.); crystalcw@foxmail.com (W.C.); yingdong333@hotmail.com (D.Y.); nsxia@xmu.edu.cn (N.X.); 2 Center for Vaccines and Immunity, The Research Institute at Nationwide Children’s Hospital, Columbus, OH 43205, USA; xyin@sbpdiscovery.org (X.Y.); charuta.ambardekar@nationwidechildrens.org (C.A.); xinlei.li@nationwidechildrens.org (X.L.); 3 State Key Laboratory of Molecular Vaccinology and Molecular Diagnostics, National Institute of Diagnostics and Vaccine Development in Infectious Diseases, School of Public Health, Xiamen University, Xiamen 361102, Fujian, China; zimintang@163.com (Z.T.); wenguiping1008@126.com (G.W.); 4 Department of Pediatrics, the Ohio State University College of Medicine, Columbus, OH 43205, USA

**Keywords:** Hepatitis E virus, neutralization assay, secreted pORF2, high throughput

## Abstract

Hepatitis E virus (HEV) is a common cause of acute hepatitis worldwide. Current methods for evaluating the neutralizing activity of HEV-specific antibodies include immunofluorescence focus assays (IFAs) and real-time PCR, which are insensitive and operationally complicated. Here, we developed a high-throughput neutralization assay by measuring secreted pORF2 levels using an HEV antigen enzyme-linked immunosorbent assay (ELISA) kit based on the highly replicating HEV genotype (gt) 3 strain Kernow. We evaluated the neutralizing activity of HEV-specific antibodies and the sera of vaccinated individuals (*n* = 15) by traditional IFA and the novel assay simultaneously. A linear regression analysis shows that there is a high degree of correlation between the two assays. Furthermore, the anti-HEV IgG levels exhibited moderate correlation with the neutralizing titers of the sera of vaccinated individuals, indicating that immunization with gt 1 can protect against gt 3 Kernow infection. We then determined specificity of the novel assay and the potential threshold of neutralizing capacity using anti-HEV IgG positive sera (*n* = 27) and anti-HEV IgG negative sera (*n* = 23). The neutralizing capacity of anti-HEV IgG positive sera was significantly stronger than that of anti-HEV IgG negative. In addition, ROC curve analysis shows that the potential threshold of neutralizing capacity of sera was 8.07, and the sensitivity and specificity of the novel assay was 88.6% and 100%, respectively. Our results suggest that the neutralization assay using the antigen ELISA kit could be a useful tool for HEV clinical research.

## 1. Introduction

Hepatitis E virus (HEV) is one of the major causes of viral acute hepatitis worldwide [1]. HEV infection is usually a self-limited acute disease but may progress to chronic hepatitis in immunocompromised patients [2]. Five genotypes (gts) have been associated with serious liver disease in humans. Gts 1 and 2 infect only humans or non-human primates and are mainly responsible for waterborne outbreaks. HEV gt 3 and 4 are zoonotic, which are transmitted mainly via contaminated meat [3]. Recently, Camelid HEV in gt 7 was found in a case of chronic infection in a liver transplant recipient, who regularly consumed camel meat and milk [4]. A gt 1 recombinant particle vaccine is highly effective for protection against gt 1 and 4 HEV infections [5]. However, no clinical studies have verified the protective effect of this vaccine against gt 3 HEV. Therefore, an accurate in vitro neutralization assay is needed to evaluate the effectiveness of neutralizing antibody responses against HEV, especially gt 3 HEV, which is widely distributed worldwide.

HEV is a single-stranded, positive-sense RNA virus. The ~7.2 kb HEV genome contains three open reading frames (ORFs). ORF1 encodes a polyprotein required for HEV replication. ORF2 encodes the capsid protein and a secreted form of ORF2 by leaky scanning, while ORF3 encodes a small protein involved in virion secretion [1,6]. The replication of HEV in vitro is inefficient; however, several strains of HEV isolated from hepatitis E patients with long-term shedding were successfully propagated in human hepatoma cell lines [7,8]. Kernow C1/p6 is a cell culture-adapted gt 3 HEV strain that replicates much faster than the parental wild-type virus, enabling evaluation of neutralizing capacities of HEV antibodies and sera in vitro [9]. Two methods are currently used to measure HEV infection: immunofluorescence focus assays (IFAs) [10,11] for detecting pORF2 expression in cells and real-time qRT-PCR [12,13,14] for detecting viral RNAs. Previous studies have demonstrated that HEV infected cells release quasi-enveloped virions into the culture supernatant and blood, and the capsid is enclosed within the host membrane [15,16]. However, recent studies have shown that the large amounts of pORF2 present in the supernatants of virus-infected cell cultures are not associated with the quasi-enveloped virions and are easily detectable by an antigen ELISA kit without detergent treatment [6,17], suggesting that the antigen in the supernatant is another potential marker that can be used to measure viral replication in cell culture.

In this study, we established an optimized high-throughput neutralization assay based on an antigen ELISA kit for detecting pORF2 secreted into the cell supernatants. This study also provides evidence that the antibody responses induced by the gt 1 vaccine can neutralize infection with gt 3 virus in vitro.

## 2. Materials and Methods

### 2.1. Cell lines, Monoclonal Antibodies, and Viruses

Huh7 cells and HepG2/C3A cells (CRL-10741, obtained from the ATCC, Manassas, VA, USA) were grown in minimum essential medium (MEM) containing 10% fetal calf serum (both from Gibco, San Diego, CA, USA) and antibiotics (100 units/mL ampicillin and 100 units/mL streptomycin) (Lukang, Shandong, China) at 37 °C with 5% CO_2_. The monoclonal antibody (mAb) 4# was donated by Youchun Wang [18]. The HEV capsid protein-specific mAb 12F12 was obtained in our laboratory using a standard murine mAb preparation protocol [19]. HEV stock was produced by transfecting Huh7 cells with in vitro-transcribed HEV Kernow C1/p6 RNA [20]. The transfected cells were cultured at 37 °C in DMEM containing 10% FBS for 15 to 20 days. Then, the transfected cells were lysed by freeze-thawing 3 times in distilled H_2_O and subjected to differential centrifugation for virus concentration [21]. The virus-containing cell lysate was centrifuged at 10,000× *g* for 30 min to remove cell debris. The virus was purified by centrifugation of the concentrated cell lysates on an isopycnic iodixanol gradient [11].

### 2.2. Blood Serum Samples

Human blood serum specimens were collected 3 years after participants received three doses of the hepatitis E vaccine. The average age of vaccinated individuals (10 Female, 5 male) was 52 ± 7.8, and they provided informed consent. Ethical approval was obtained from the Research Ethics Review Committee of Xiamen University. The anti-HEV IgG level of human sera were determined using a WHO reference serum [22]. The IgG levels in the human sera were greater than 0.077 WU (WHO units of anti-HEV IgG) per milliliter (WE-7296; Wantai Biopharm, Beijing, China) [5,23]. Twelve serum samples from three HEV-infected rhesus macaques were included to evaluate the neutralizing capacities. Three rhesus macaques were inoculated intravenously with 5 × 10^5^ of human HEV gt 1 (strain Xinjiang) and 3 (strain JRC-HE3). Specifically, macaques 1 and 2 were inoculated with HEV gt 1 and macaque 3 was inoculated with HEV gt3. Blood was collected twice a week. Eight serum samples from HEV negative individuals, eight serum samples from patients with Cytomegalovirus (CMV), seven serum samples from patients with Epstein-Barr virus (EBV) were also included to analyze the specificity of the neutralizing assay. Hepatitis E negative individuals were defined as individuals who were negative for HEV IgM, IgG, antigen, and RNA. CMV-infected patients were defined as patients who were CMV IgG positive and negative for HEV markers. EBV-infected patients were defined as patients who were EBV IgG positive and negative for HEV markers. All of the sera were stored at −20 °C. The sera for the neutralization assay were heat-inactivated at 56 °C for 30 min.

### 2.3. Real-Time PCR

Viral RNA from culture supernatants and lysates was extracted with the GenMag virus DNA/RNA isolation Kit (Genmag, Beijing, China) in accordance with the manufacturer’s instructions. Real-time PCR was performed to quantify the copy numbers of HEV RNA as previously reported [24]. A CFX96 real-time system and C1000 thermocycler device (Bio-Rad, Inc., Hercules, CA, USA) were used for all real-time PCR tests. For generation of standard quantitation curves, the threshold cycle (CT) values were plotted as a function of the input HEV viral copy numbers. The copy numbers were determined by calibrating the instrument with different concentrations of the plasmid standard.

### 2.4. Virus Infection

HepG2/C3A cells (3 × 10^4^) were seeded onto 96-well plates (PerkinElmer, Inc., Waltham, MA, USA) a day before infection. Cells were inoculated with HEV at 30 MOI (30 HEV genome equivalents per cell) for 6 h at 37 °C, washed three times with PBS, and refed with fresh medium supplemented with 10% FBS, 2% DMSO, 100 U penicillin mL^−1^, 0.1 mg streptomycin mL^−1^, and 0.1 mg gentamicin mL^−1^, followed by incubation at 37 °C for 2–10 days.

### 2.5. Neutralization Assay

HepG2/C3A cells were seeded onto 96-well plates (PerkinElmer, Inc., Waltham, MA, USA) at a concentration of 3 × 10^4^ cells per well a day before infection. Mixtures of purified virus and serial four-fold dilutions (beginning with a dilution of 1:3) of sera or serial five-fold dilutions (beginning with 500 μg/mL) of mAb 12F12 were incubated for 30 min at 37 °C. Then the mixtures were inoculated with cell monolayer for 6h at 37 °C, washed 3 times with PBS, and refed with fresh medium supplemented with 10% FBS, 2% DMSO, 100 U penicillin mL^−1^, 0.1 mg streptomycin mL^−1^, and 0.1 mg gentamicin mL^−1^, followed by incubation at 37 °C for 8 days.

### 2.6. Immunofluorescence Assay

Cells were washed once and fixed with 4% paraformaldehyde (Sigma Aldrich, St. Louis, MO, USA) for 30 min and permeabilized with 0.3% Triton X-100 (Amresco) for 15 min in PBS. After blocking in 2% goat serum diluted in 2% BSA for 1 h, cells were incubated with mAb 4# for 1 h and labeled with fluorescein isothiocyanate-conjugated goat anti-mouse IgG (Molecular Probes) for 1 h. After adding DAPI (4,6-Diamidino-2-Phenylindole, Dihydrochloride) (Invitrogen, Carlsbad, CA, USA). The stained cells were visualized with Opera Phenix (PerkinElmer, Inc., Waltham, MA, USA) and automatically counted.

### 2.7. Detection of HEV Antigen

The antigen in the cell supernatants and cell lysates was detected with the Wantai (Wantai Biopharm, Beijing, China) HEV Antigen ELISA Kit according to the manufacturer’s instructions.

### 2.8. Statistical Analysis

Non-linear regression, linear regression, and *t*-test were performed using GraphPad Prism (GraphPad, San Diego, CA, USA). Non-linear regression (log [inhibitor] vs. response, variable slope) was used to obtain IC50 and ID50 in neutralizing assays. Linear regression was conducted to analyze the correlation between the IFA and ELISA results and the correlation between IgG levels and the IFA and ELISA results. A paired *t*-test was used to compare neutralization titers obtained by neutralizing assay involving IFA and antigen ELISA test, and *p* < 0.05 was considered a significant difference. Unpaired Mann-Whitney *t* test was conducted to obtain *p* values between groups of anti-HEV positive and anti-HEV IgG negative sera. ROC curve analysis using SPSS was used to determine the potential decisive threshold of neutralizing capacity.

## 3. Results

### 3.1. Comparing the Different Methods for Measuring Kernow C1-p6 Infection in HepG2/C3A Cells

**Figure 1:**
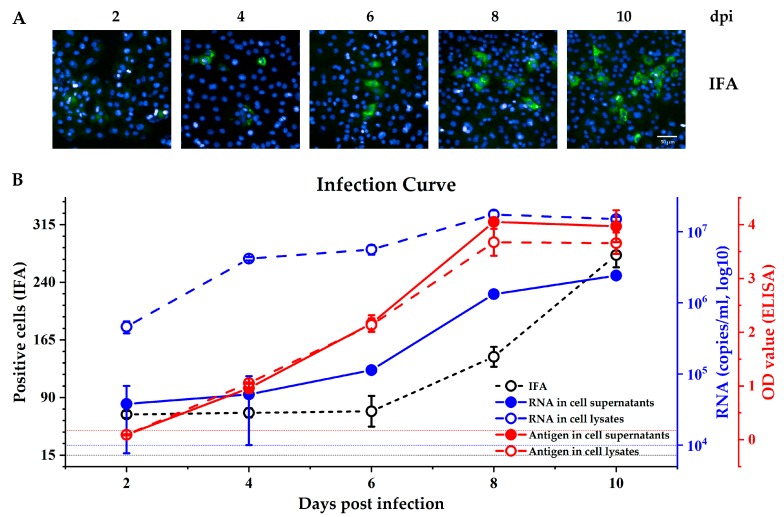
Hepatitis E virus (HEV) infection kinetics in HepG2/C3A cells. (**A**) Immunofluorescence images show the expression of pORF2 (green) at different times after inoculation. The scale bar represents 50 μm. (**B**) The number of positive cells determined by immunofluorescence focus assay (IFA) is shown as a black line. The black dashed line represents average positive cells in the negative controls. HEV antigen pORF2 in the supernatant and cell lysate was detected by an HEV antigen test (red lines). The red dashed line (OD value = 0.17) represents the cut-off value of the negative controls. HEV RNA in the supernatants and cell lysates of HEV-infected HepG2 culture (blue lines). The blue short dashed line indicates the qRT-PCR detection limit. The results represent the means ± SEMs of four independent experiments.

A previous report showed that an antigen ELISA test is effective at detecting secreted pORF2 [6]. Therefore, we used this test to measure pORF2 levels in both the culture supernatant and cell lysate of virus-infected cells and compared the results with those of IFA and real-time PCR detection. As shown in Figure 1, the antigens were detectable at 4 days post infection (dpi) (OD ≈ 1), and the OD value was >3 in both the supernatant and cell lysate from the 6th day post inoculation. The values from the antigen tests were stable among different repeat wells (narrow SEM range), and the readout was similar in the culture supernatant and cell lysate at each time point. However, the average RNA level in the cell lysate were higher than those in the culture supernatant (four independent experiments at each indicated time point). The RNA copy number in the cell lysate was 4.6 × 10^5^ at 2 dpi and reached 10^7^ at 8 dpi The RNA was also detectable in the supernatant at 2–4 dpi, but the results fluctuated with a wide range from 10^4^ to 10^5^. A stable RNA level was detected in the supernatant at 6 dpi, when the significant levels of the antigen could also be detected. The IFA results showed that the pORF2 expressed in cells could be detected at 2 dpi, but the fluorescence signal was unclear (Figure 1A, 2 dpi). The signal gradually increased from 2 dpi to 6 dpi, but the number of positive cells increased until 8 dpi (Figure 1B, black line). On average, 140 and 270 positive cells were counted at 8 dpi and 10 dpi, and these values were higher than those observed at 2–6 dpi However, considering that 5 × 10^4^ cells were seeded per well before infection, the rate of occurrence of positive cells remained low. These results indicated that detection of the antigen secreted in the culture supernatant by ELISA was a highly sensitive method for measurement of HEV infection in vitro, and 6–8 dpi was found to be the optimal time point for detection; subsequent assays were incubated for this duration.

### 3.2. Neutralization Assay with a HEV-Specific mAb against Kernow C1 p6 Strain Analyzed by IFA and ELISA

**Figure 2:**
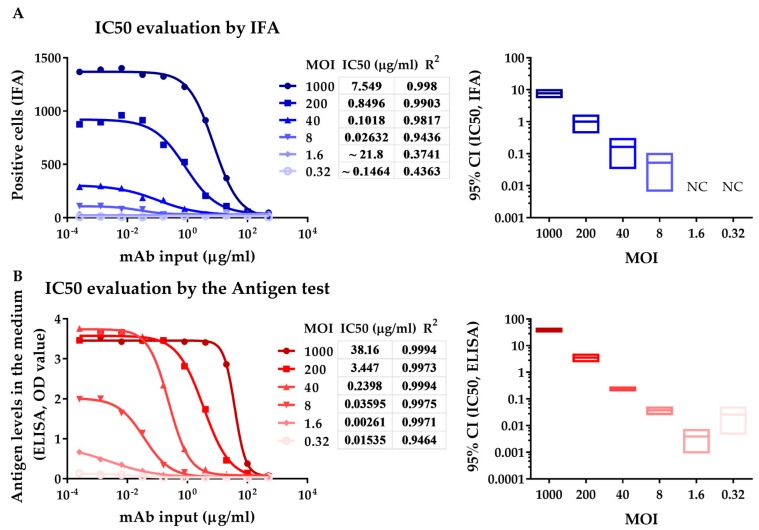
Comparison of the IFA and enzyme-linked immunosorbent assay (ELISA) methods by measuring the neutralizing capacity of the HEV-specific mAb 12F12. (**A**) IC50 evaluation by IFA shows the number of positive cells 8 days after inoculation with mixtures of dilutions of 12F12 and HEV (0.32 to 1000 MOI) (left panel). (**B**) IC50 evaluation by neutralizing assay involving antigen ELISA test shows the antigen in medium 8 days after inoculated with mixtures of dilutions of 12F12 and HEV (0.32 to 1000 MOI) (left panel). The left panel curves were fitted for nonlinear regression (log [inhibitor] vs. response, variable slope). The right panels display 95% confidence intervals of IC50 at various MOIs detected by IFA and ELISA. NC, not calculated.

Using the neutralizing assay involving antigen ELISA test, the neutralizing ability of antibodies or sera was evaluated by detecting secreted pORF2 in culture supernatant, which is associated with virus replication. Neutralizing ability was evaluated by measuring the number of infected cells using IFA. To determine the optimal range of viral input, we performed a neutralization assay at MOIs ranging from 0.32 to 1000. IFA and antigen ELISA test were conducted simultaneously at 8 dpi. The mAb 12F12 is an HEV-specific potent neutralizing mAb. The IC50 values measured by the antigen test were almost equal to those detected by IFA at MOIs from 8 to 40. The IC50s of 12F12 detected by both IFA and the antigen test increased with increasing viral input (Figure 2), which was likely due to the increasing dose of the virus. Reliable IC50s could be obtained by the IFA at MOIs ranging from 40 to 1000 (R^2^ > 0.98) (Figure 2A). However, the IC50 values obtained at MOIs from 0.32 to 8 were unreliable (wide 95% CI range), which was possibly caused by the weak infection of cells (approximately 25, the background of positive cells had a value of approximately 20). The IC50 of 12F12 was measurable by the antigen test even at an MOI of 0.32, although the degree of fit (R^2^ < 0.95) was not sufficient. Accurate and reliable IC50s could be obtained at MOIs ranging from 8 to 40 (R^2^ > 0.99, narrow 95% CI range, Figure 2B). At MOIs from 0.32 to 1.6, the OD value was not within the linear range of the ELISA kit, which likely explains the relatively wide range of the 95% CI value of IC50 and the variability of IC50 at an MOI of 0.32. At MOIs from 200 to 1000, the OD value exceeded the upper limit of HEV antigen test, which likely accounted for the underestimated IC50 of 12F12. (Figure 2B). These results indicated that reliable IC50s could be obtained by neutralizing assay involving antigen ELISA test at MOIs from 8 to 40, while the neutralizing assay involving IFA was at MOI from 40 to 1000, which again demonstrates antigen ELISA test was more sensitive than IFA at detecting virus replication in a neutralizing assay.

### 3.3. Neutralization of Human Sera after Gt 1 HEV Vaccine Administration against Gt 3 HEV (Kernow)

**Figure 3:**
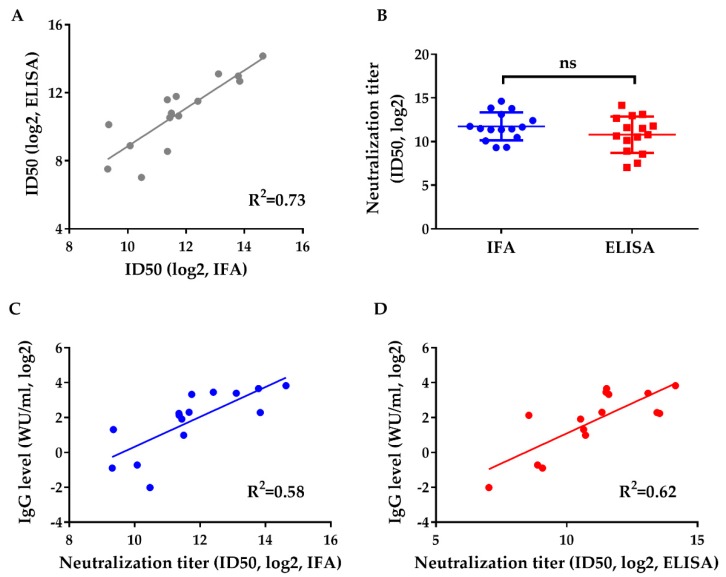
Neutralization test of sera of vaccinated subjects evaluated by IFA and ELISA and the relationship between neutralization titer and IgG level. The neutralizing capacities against the Kernow of vaccinated human sera (*n* = 15) were measured by IFA and ELISA. The IgG levels of human sera were all above the 0.077 WU per milliliter. (**A**) Correlation of the neutralization titers measured by IFA and ELISA. (**B**) Comparison of the neutralization titers measured by IFA and ELISA using a paired *t*-test. Two-sided *p*-values are given; “ns” indicates that there were no significant differences. (**C**) Correlation analysis between IgG level and the neutralization titer detected by IFA. (**D**) Correlation analysis between IgG level and the neutralization titer detected by ELISA. The neutralization titers of the sera were calculated with 50% inhibitory dilutions (ID50 values). The results were log2-transformed.

The method was then used to evaluate the neutralizing capacity of human sera from HEV vaccinated subjects. Previous research has shown that p239 derived from gt 1 protects against gt 1 and gt 4 infection with high efficacy [5]. However, no clinical studies have verified the protective effects of p239 against gt 3. Therefore, we collected serum samples from participants who had been subjected to vaccine administration (*n* = 15) to evaluate their neutralizing capacity and the consistency between the IFA and ELISA detection methods. Serial 4-fold dilutions of human sera were inoculated with virus at an MOI of 20 for 30 min before incubating with HepG2 cells for 6 h. The neutralizing titer was determined at 8 dpi. The infectivity of Kernow was inhibited effectively by the sera of vaccinated individuals (Figure 3). Figure 3A shows the correlation analysis of neutralizing titers evaluated by IFA and ELISA. A good linear relationship was observed between the two assays (R^2^ = 0.73), and the ID50 values measured by the ELISA were almost equal to those determined by IFA. Additionally, there was no statistically significant difference between the ID50 values measured by IFA and ELISA (Figure 3B). Furthermore, a moderate correlation was observed between IgG levels and the neutralization titers measured by IFA (R^2^ = 0.58) and ELISA (R^2^ = 0.62) (Figure 3C,D). The slight increase in R^2^ detected by ELISA likely contributes to the high sensitivity of this method at low IgG levels, when weak infection could be measured accurately by ELISA. These results support the hypothesis that the gt1 HEV vaccine could protect against gt 3 infection.

### 3.4. Determination of the Potential Threshold for Neutralizing Capacity and Specificity of Neutralizing Assay Involving Detection of Secreted pORF2

**Figure 4:**
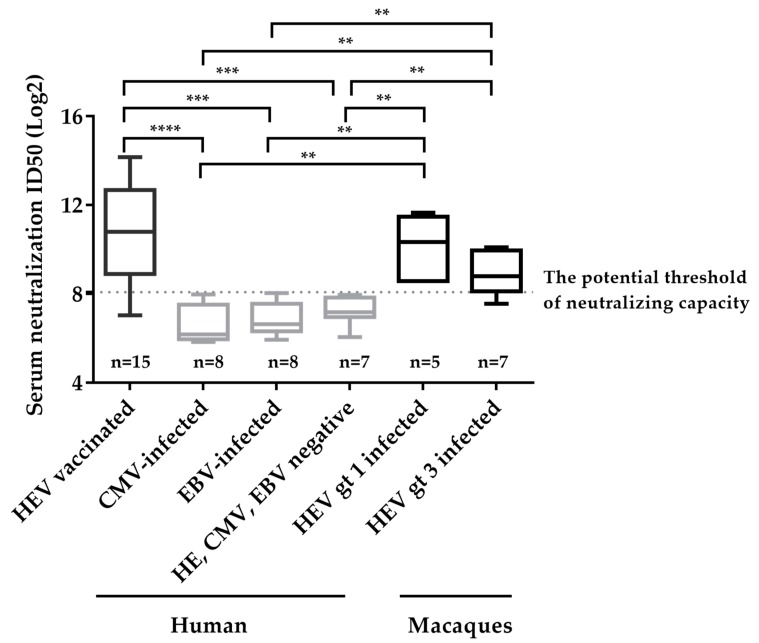
Determination of the potential threshold for neutralizing capacity and specificity of the neutralizing assay involving detection of secreted pORF2. Sera collected from 8 non-HEV, 8 CMV-infected individuals, 8 EBV-infected individuals and 15 HEV vaccinated donors, and 5 sera collected from HEV gt 1 infected macaques and 7 sera collected from HEV gt 3 infected macaques were included to analyze the specificity of the assay. The non-HEV sera were defined as HEV RNA negative, anti-HEV antibodies negative and HEV antigen negative; The CMV-infected patients were defined as anti-CMV IgG positive and HEV markers negative; The EBV-infected patients were defined as anti-EBV IgG positive and HEV markers negative. The potential threshold was set at maximum of Youden index (8.07). The dotted line represents the threshold of the neutralizing capacity of sera. The detection results for each group are shown as the range (whiskers), interquartile range (boxes), and median (line within boxes). **, *p* < 0.01; ***, *p* < 0.001; ****, *p* < 0.0001.

We then applied the neutralizing assay on some other anti-HEV IgG negative and positive sera, including sera from CMV infected individuals (anti-CMV IgG positive, anti-HEV IgG negative, *n* = 8), EBV infected individuals (anti-EBV positive, anti-HEV negative, *n* = 7), healthy donors (anti-CMV, EBV, HEV IgG negative, *n* = 8), and HEV-infected macaques (gt 1 HEV infected, *n* = 5; gt 3 HEV infected, *n* = 7) (Table S1), and integrated these data with that from HEV vaccinated donors to determine the potential threshold of neutralizing capacity and specificity of the novel neutralizing assay. As shown in Figure 4, the neutralization titers of anti-HEV IgG positive groups were significantly higher than those of anti-HEV IgG negative groups. An ROC (Receiver Operating Characteristic) curve analysis was further used to obtain a potential threshold of neutralizing capacity using ID50s from HEV IgG positive groups (*n* = 27) and HEV IgG negative groups (*n* = 23). The area under the ROC curve was 0.963, indicating high evaluation value of the novel assay (Figure S1). When the threshold was set to 8.07, the maximum of Youden Index (Youden Index = Sensitivity + Specificity – 1), the calculated sensitivity and specificity were 88.9% and 100%, respectively (Figure S2, Table S2). Three of twenty-seven HEV IgG positive sera were negative for neutralizing capacity, which was likely due to low IgG level, and all HEV-IgG negative sera were negative for neutralizing capacity (Figure 4). These results indicates our assay is valuable in evaluating neutralizing ability.

## 4. Discussion

The inefficient propagation of HEV in cell models has posed many challenges to understand the mechanism of virus infection and hindered the ability to evaluate the neutralization of sera and antibodies in a high-throughput manner. In this study, we developed a neutralization model that could be detected by an antigen kit, which improved the sensitivity of infection detection. In the quantification of Kernow infectivity using qRT-PCR, IFA, and ELISA, definite positive values were detected by antigen testing, which yielded results 4 d earlier than IFA, and ELISA exhibited better reproducibility in duplicated cultures than qRT-PCR. A previous study showed that the HEV-secreted antigen pORF2 was associated with viral replication but was translated more efficiently than the HEV capsid antigen [6], which might explain the high sensitivity of the HEV antigen ELISA kit. However, the neutralization titer was virus dose dependent. Therefore, viral input at MOIs from 8 to 40 is recommended to maintain the IC50 values within a normal range.

Using this method, we evaluated the neutralizing titers of sera from vaccinated subjects. A linear regression analysis indicated a high degree of correlation between the neutralizing titers measured by IFA and ELISA. Furthermore, a correlation between the IgG level and the neutralizing titers measured by ELISA and IFA was observed, indicating that gt 1 vaccination effectively neutralized the HEV gt 3 strain Kernow, which further supports the hypothesis that the hepatitis E gt 1 vaccine might protect against infection by different HEV gts.

However, this study also showed that the ID50 measured by the antigen test was slightly lower than that measured by the IFA at low neutralizing titers, which might be due to the high sensitivity and relatively low upper threshold of detection of the antigen test, but this discrepancy can be addressed by diluting the culture supernatant samples before detection or by decreasing the viral input. Additionally, the IgG levels and neutralizing titers were not always correlated. The immune response of vaccinated individuals could vary, or the IgM response may be associated with the neutralizing titer. Further studies are needed to provide insight into these aspects.

We determined the potential threshold of neutralizing capacity using ROC curve analysis. Despite limited number of samples (anti-HEV IgG positive, *n* = 27; anti-HEV negative, *n* = 23), the novel assay with this threshold showed a good specificity (100%). However, the neutralization titer of three sera with low IgG level was below the threshold, which is likely because the sensitivity of the neutralizing assay was lower than that of HEV IgG ELISA kit. In addition, sera of both gt 1- and gt 3-infected macaques neutralized HEV effectively, which is not affected by virions in serum, suggesting the novel assay is valuable in evaluating neutralizing capacity.

However, the novel neutralization assay was based on gt 3 Kernow C1/p6 because this strain replicates much faster than the parental wild-type virus [20]. Since the antigen ELISA test had similar detection abilities for viruses with different genotypes [25] and the production of secreted pORF2 of HEV is evolutionary conservative [6], the antigen ELISA test will also be helpful in establishment of neutralization assay based on HEV gt 1, gt 2, gt 4, and newly discovered gt 7 stains.

We evaluated neutralizing capacity using nonenveloped virions rather than quasi-enveloped form of HEV (eHEV), because eHEV is insensitive to neutralizing antibodies [14]. However, given the increasing concerns about persistent HEV infection and its potential for transfusion mediated transmission [26,27], the mechanism of eHEV neutralization is of further interest.

In summary, we have shown that the neutralization assay involving detection of secreted pORF2 can be used as an alternative method for evaluating the neutralizing capacity of anti-HEV samples in high-throughput. This assay is also applicable for HEV antibodies and drug screening studies.

## Supplementary Materials

The following are available online at http://www.mdpi.com/1999-4915/11/1/64/s1, Figure S1: An ROC curve of neutralization titers of sera (anti-HEV IgG positive, *n* = 27; anti-HEV IgG negative, *n* = 23) displayed, Figure S2: Curves of Youden index, specificity, sentivity changed with the threshold of neutralizing capacity of sera. Youden Index = Sensitivity + Specificity − 1, Table S1: Serum samples obtained from HEV-infected macaques, Table S2: Sensitivity and Specificity of threshold of neutralizing capacity.
