# Supplementary material for: An Optimized High-Throughput Neutralization Assay for Hepatitis E Virus (HEV) Involving Detection of Secreted Porf2
Source: Viruses. 2019 Jan 15;11(1):64. doi: 10.3390/v11010064 (PMC6356577; doi:10.3390/v11010064)
Supplement: Supplementary file 1 [file viruses-11-00064-s001.zip › Fig in sup/supplementary figures.docx]

**Supplementary Figures**

An optimized high-throughput neutralization assay for hepatitis E virus (HEV) involving detection of secreted pORF2

Chang Liu^1,^ †, Wei Cai^1,^ †, Xin Yin^2,^ †, Zimin Tang^3^, Guiping Wen^3^, Charuta Ambardekar^2^, Xinlei Li^2^, Dong Ying^1^, Zongdi Feng^2, 4,^ *, Zizheng Zheng^3,^ *, Ningshao Xia^1, 3^

^1^ State Key Laboratory of Molecular Vaccinology and Molecular Diagnostics, National Institute of Diagnostics and Vaccine Development in Infectious Diseases, School of Life Sciences, Xiamen University, Xiamen, Fujian, PR China; [changl@stu.xmu.edu.cn](mailto:changl@stu.xmu.edu.cn); [crystalcw@foxmail.com](mailto:crystalcw@foxmail.com); [yingdong333@hotmail.com](mailto:yingdong333@hotmail.com); [nsxia@xmu.edu.cn](mailto:nsxia@xmu.edu.cn)

^2^ Center for Vaccines and Immunity, The Research Institute at Nationwide Children’s Hospital, Columbus, OH 43205, USA; [xyin@sbpdiscovery.org](mailto:xyin@sbpdiscovery.org); [charuta.ambardekar@nationwidechildrens.org](mailto:charuta.ambardekar@nationwidechildrens.org); [xinlei.li@nationwidechildrens.org](mailto:xinlei.li@nationwidechildrens.org)

^3^ State Key Laboratory of Molecular Vaccinology and Molecular Diagnostics, National Institute of Diagnostics and Vaccine Development in Infectious Diseases, School of Public Health, Xiamen University, Xiamen, Fujian, PR China; [zimintang@163.com](mailto:zimintang@163.com); [wenguiping1008@126.com](mailto:wenguiping1008@126.com); [nsxia@xmu.edu.cn](mailto:nsxia@xmu.edu.cn)

^4^ Department of Pediatrics, the Ohio State University College of Medicine, Columbus, OH 43205, USA

***** Correspondence: [Zongdi.Feng@nationwidechildrens.org](mailto:Zongdi.Feng@nationwidechildrens.org), [zhengzizheng@xmu.edu.cn](mailto:zhengzizheng@xmu.edu.cn)

† The authors contributed equally to this paper.


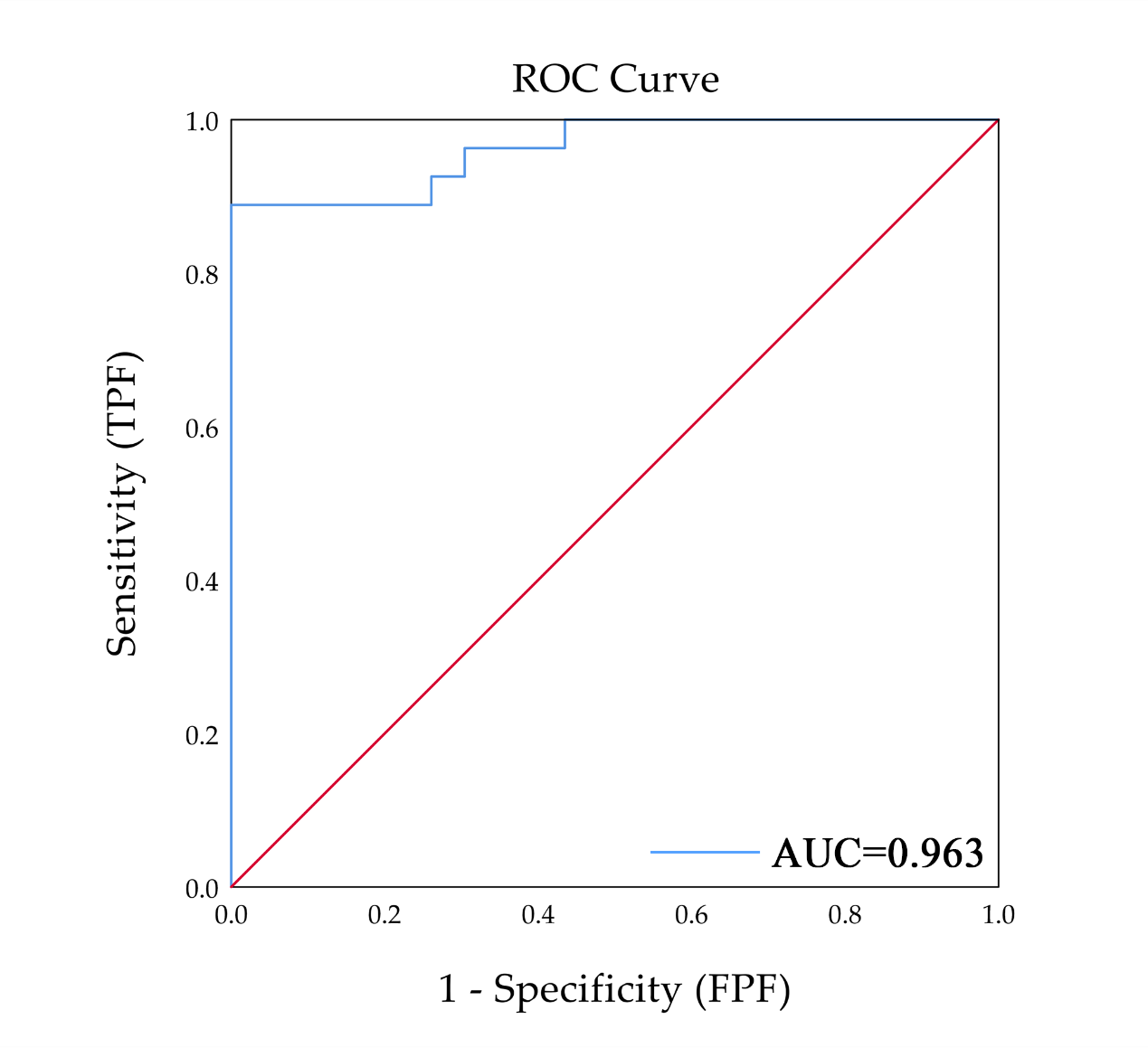
Figure 1. An ROC curve of neutralization titers of sera (anti-HEV IgG positive, n = 27; anti-HEV IgG negative, n = 23) displayed. ROC, receiver operating characteristic; AUC, area under the curve.


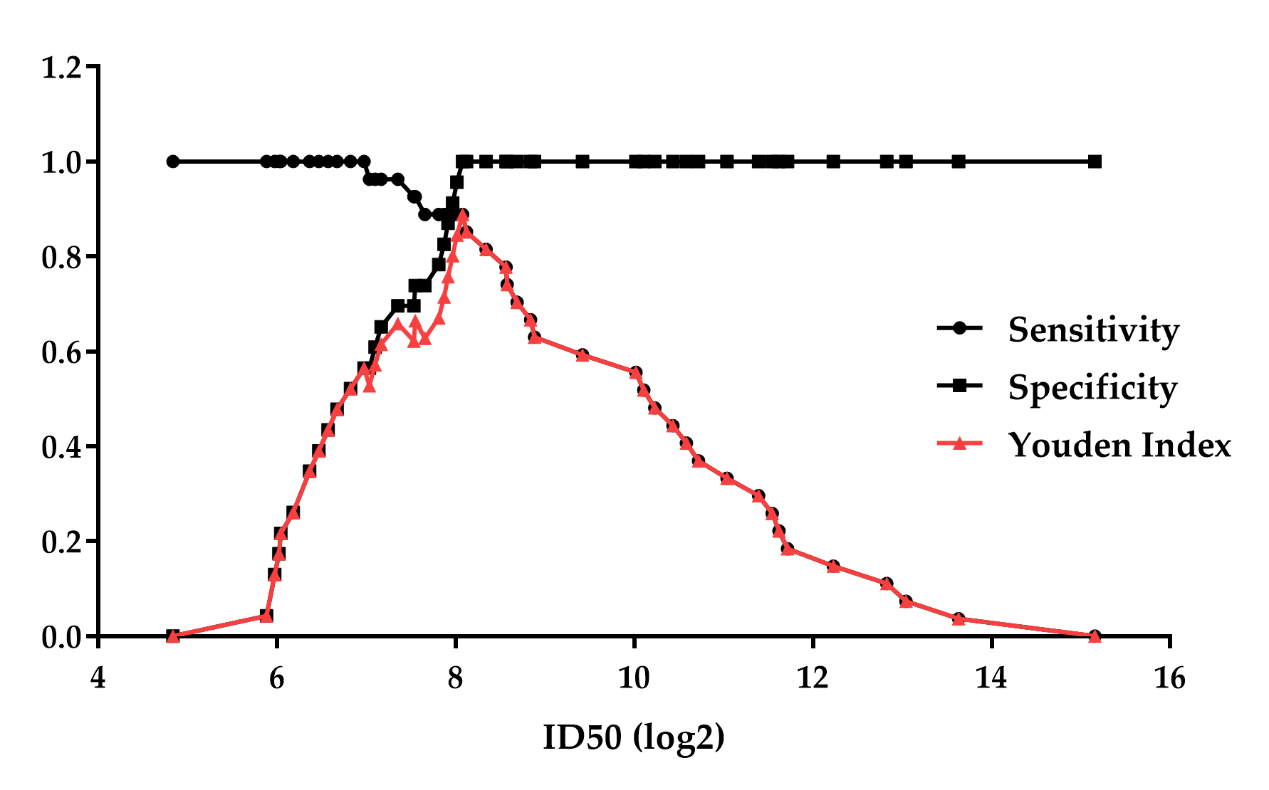


Figure 2. Curves of Youden index, specificity, sentivity changed with the threshold of neutralizing capacity of sera. Youden Index=Sensitivity+Specificity-1.
