# Supplementary material for: An Optimized High-Throughput Neutralization Assay for Hepatitis E Virus (HEV) Involving Detection of Secreted Porf2
Source: Viruses. 2019 Jan 15;11(1):64. doi: 10.3390/v11010064 (PMC6356577; doi:10.3390/v11010064)
Supplement: Supplementary file 1 [file viruses-11-00064-s001.zip › Fig in sup/Table 1.docx]

| Table 1. Serum samples obtained from HEV-infected macaques | | | | |
| --- | --- | --- | --- | --- |
| Macaque no. | HEV genotype | IgG (S/CO)a | IgM (S/CO) | HEV RNA titer (copies/mL) |
| 1 | 1 | 10.58 | 15.02 | 2.3×106 |
| 1 | 1 | 23.95 | 14.61 | 1.7×106 |
| 1 | 1 | 200.05 | 17.09 | - |
| 1 | 1 | 274.90 | 14.62 | - |
| 2 | 1 | 349.90 | 2.06 | - |
| 2 | 1 | 811.24 | 1.87 | - |
| 3 | 3 | 15.38 | 0.03 | 2.8×106 |
| 3 | 3 | 7.16 | 0.02 | 8.1×106 |
| 3 | 3 | 55.04 | 0.03 | 1.7×106 |
| 3 | 3 | 102.37 | 0.04 | 3.5×105 |
| 3 | 3 | 145.92 | 0.04 | - |
| 3 | 3 | 137.45 | 0.04 | - |
| a S/CO represents the ratio between OD value of sample and cutoff value, negative (S/CO<1) for IgM. | | | | |
