# Supplementary material for: An Optimized High-Throughput Neutralization Assay for Hepatitis E Virus (HEV) Involving Detection of Secreted Porf2
Source: Viruses. 2019 Jan 15;11(1):64. doi: 10.3390/v11010064 (PMC6356577; doi:10.3390/v11010064)
Supplement: Supplementary file 1 [file viruses-11-00064-s001.zip › Fig in sup/Table 2.docx]

**Table 2. Sensitivity and Specificity of threshold of neutralizing capacity**

| **Positive if Greater Than or Equal toa** | **Sensitivity** | **1 - Specificity** | **Youden Indexb** |
| --- | --- | --- | --- |
| 4.8332 | 1 | 1 | 0 |
| 5.8834 | 1 | 0.957 | 0.043 |
| 5.9744 | 1 | 0.87 | 0.13 |
| 6.0264 | 1 | 0.826 | 0.174 |
| 6.0438 | 1 | 0.783 | 0.217 |
| 6.1817 | 1 | 0.739 | 0.261 |
| 6.3649 | 1 | 0.652 | 0.348 |
| 6.4753 | 1 | 0.609 | 0.391 |
| 6.5772 | 1 | 0.565 | 0.435 |
| 6.6614 | 1 | 0.522 | 0.478 |
| 6.8148 | 1 | 0.478 | 0.522 |
| 6.9753 | 1 | 0.435 | 0.565 |
| 7.035 | 0.963 | 0.435 | 0.528 |
| 7.0966 | 0.963 | 0.391 | 0.572 |
| 7.1668 | 0.963 | 0.348 | 0.615 |
| 7.3536 | 0.963 | 0.304 | 0.659 |
| 7.531 | 0.926 | 0.304 | 0.622 |
| 7.5471 | 0.926 | 0.261 | 0.665 |
| 7.6544 | 0.889 | 0.261 | 0.628 |
| 7.8114 | 0.889 | 0.217 | 0.672 |
| 7.8725 | 0.889 | 0.174 | 0.715 |
| 7.916 | 0.889 | 0.13 | 0.759 |
| 7.966 | 0.889 | 0.087 | 0.802 |
| 8.0073 | 0.889 | 0.043 | 0.846 |
| 8.0745 | 0.889 | 0 | 0.889 |
| 8.1203 | 0.852 | 0 | 0.852 |
| 8.341 | 0.815 | 0 | 0.815 |
| 8.5634 | 0.778 | 0 | 0.778 |
| 8.576 | 0.741 | 0 | 0.741 |
| 8.6889 | 0.704 | 0 | 0.704 |
| 8.84 | 0.667 | 0 | 0.667 |
| 8.8831 | 0.63 | 0 | 0.63 |
| 9.4217 | 0.593 | 0 | 0.593 |
| 10.0196 | 0.556 | 0 | 0.556 |
| 10.1042 | 0.519 | 0 | 0.519 |
| 10.2288 | 0.481 | 0 | 0.481 |
| 10.431 | 0.444 | 0 | 0.444 |
| 10.5854 | 0.407 | 0 | 0.407 |
| 10.7172 | 0.37 | 0 | 0.37 |
| 11.0385 | 0.333 | 0 | 0.333 |
| 11.3914 | 0.296 | 0 | 0.296 |
| 11.5446 | 0.259 | 0 | 0.259 |
| 11.62 | 0.222 | 0 | 0.222 |
| 11.7164 | 0.185 | 0 | 0.185 |
| 12.228 | 0.148 | 0 | 0.148 |
| 12.8268 | 0.111 | 0 | 0.111 |
| 13.0428 | 0.074 | 0 | 0.074 |
| 13.6334 | 0.037 | 0 | 0.037 |
| 15.159 | 0 | 0 | 0 |

a The smallest threshold value is the minimum observed test value minus 1, and the largest threshold value is the maximum observed test value plus 1. All the other threshold values are the averages of two consecutive ordered observed test values. b Youden Index = TPF - FPF，TPF = Sensitivity，FPF = 1 - Specificity
