# Supplementary figures and images for: An Optimized High-Throughput Neutralization Assay for Hepatitis E Virus (HEV) Involving Detection of Secreted Porf2
Source: Viruses. 2019 Jan 15;11(1):64. doi: 10.3390/v11010064 (PMC6356577; doi:10.3390/v11010064)

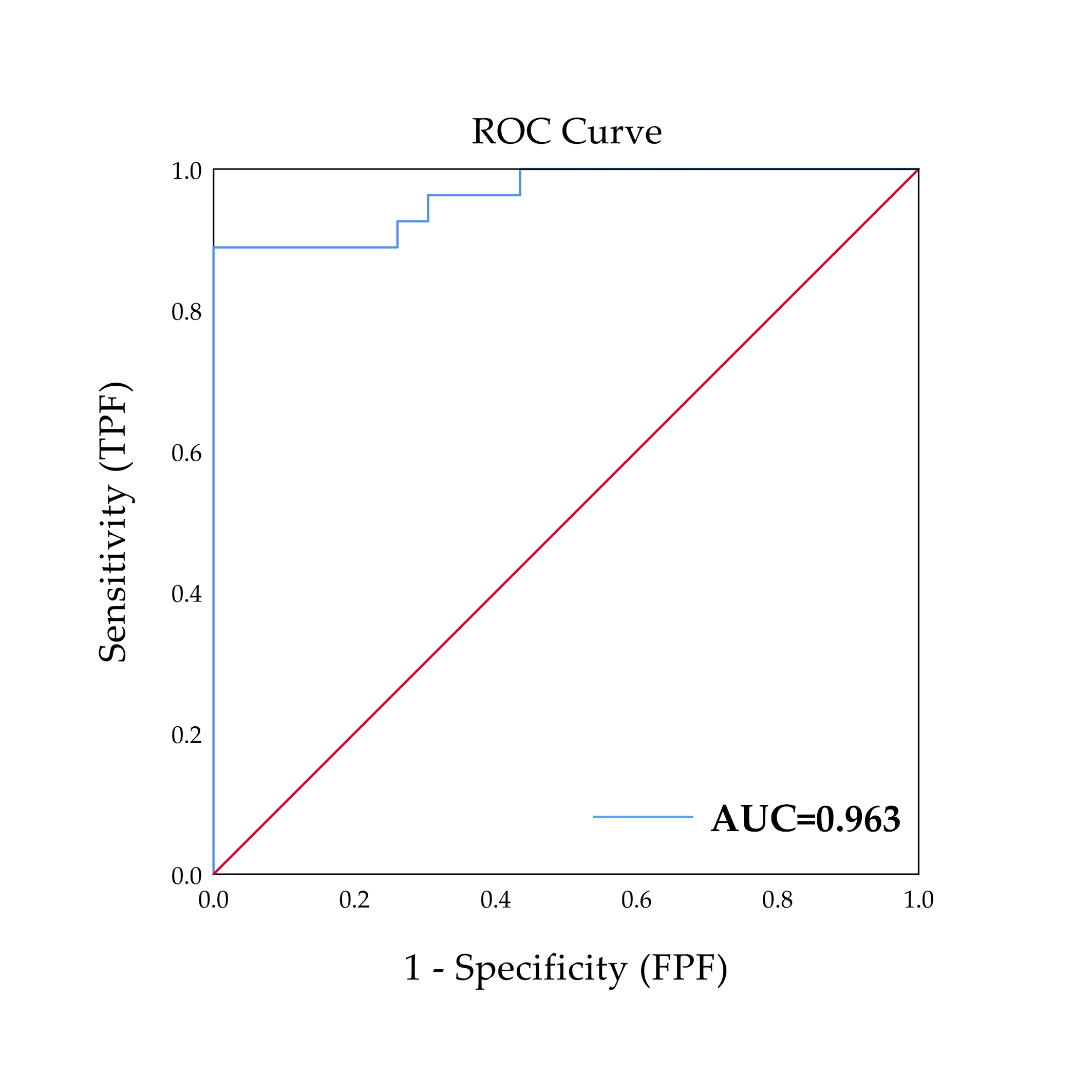

Supplement: Supplementary file 1 [file viruses-11-00064-s001.zip › Fig in sup/Fig 1.tif]

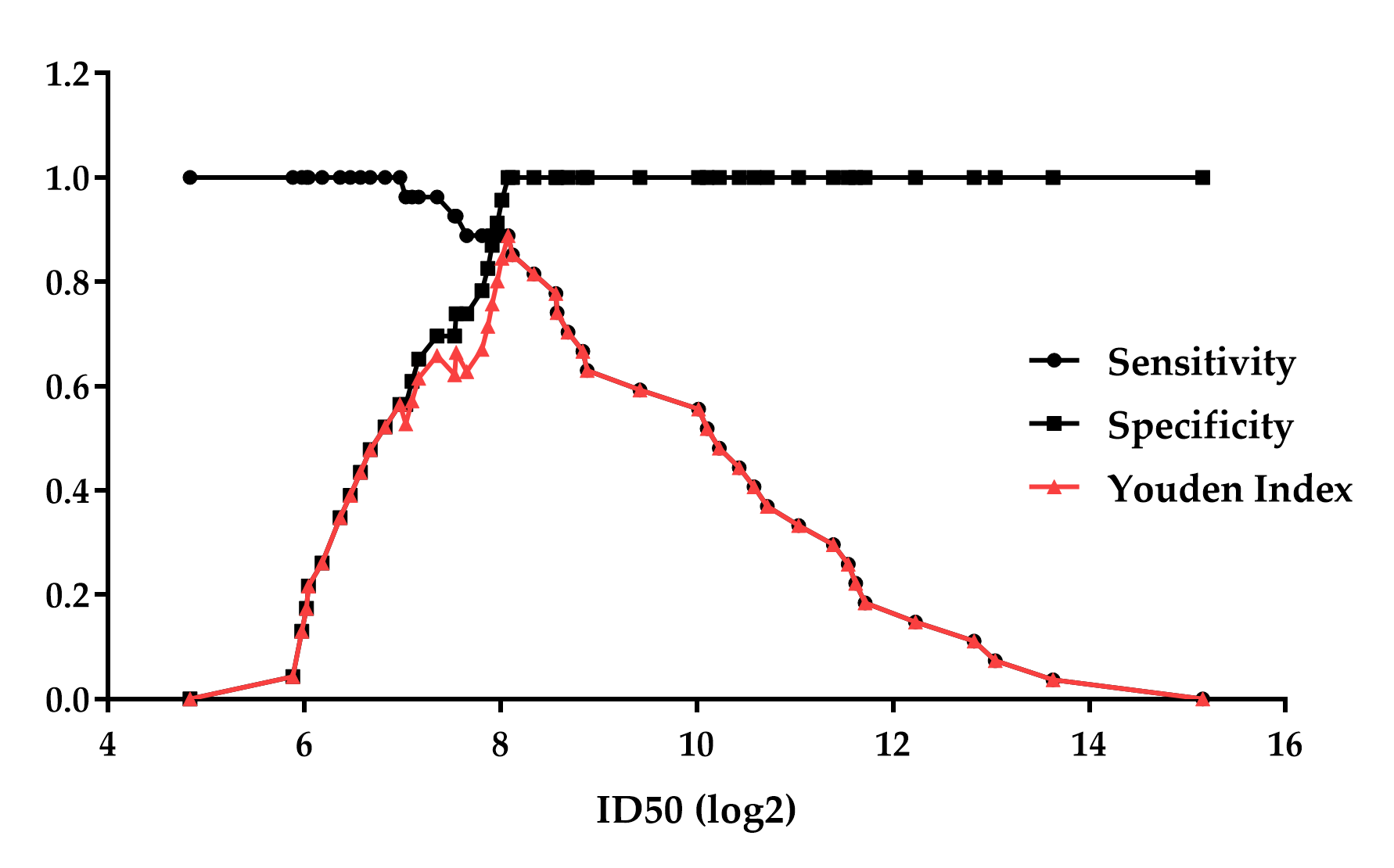

Supplement: Supplementary file 1 [file viruses-11-00064-s001.zip › Fig in sup/Fig 2.tif]
